# Developing community-based preventive interventions in Hong Kong: a description of the first phase of the family project

**DOI:** 10.1186/1471-2458-12-106

**Published:** 2012-02-07

**Authors:** Sunita M Stewart, Cecilia S Fabrizio, Malia R Hirschmann, Tai Hing Lam

**Affiliations:** 1 University of Texas Southwestern Medical Center at Dallas, Texas, USA; 2 The University of Hong Kong, Pokfulam, Hong Kong, SAR

## Abstract

**Background:**

This paper describes the development of culturally-appropriate family-based interventions and their relevant measures, to promote family health, happiness and harmony in Hong Kong. Programs were developed in the community, using a collaborative approach with community partners. The development process, challenges, and the lessons learned are described. This experience may be of interest to the scientific community as there is little information currently available about community-based development of brief interventions with local validity in cultures outside the West.

**Methods:**

The academic-community collaborative team each brought strengths to the development process and determined the targets for intervention (parent-child relationships). Information from expert advisors and stakeholder discussion groups was collected and utilized to define the sources of stress in parent-child relationships.

**Results:**

Themes emerged from the literature and discussion groups that guided the content of the intervention. Projects emphasized features that were appropriate for this cultural group and promoted potential for sustainability, so that the programs might eventually be implemented at a population-wide level. Challenges included ensuring local direction, relevance and acceptability for the intervention content, engaging participants and enhancing motivation to make behavior changes after a brief program, measurement of behavior changes, and developing an equal partner relationship between academic and community staff.

**Conclusions:**

This work has public health significance because of the global importance of parent-child relationships as a risk-factor for many outcomes in adulthood, the need to develop interventions with strong evidence of effectiveness to populations outside the West, the potential application of our interventions to universal populations, and characteristics of the interventions that promote dissemination, including minimal additional costs for delivery by community agencies, and high acceptability to participants.

## Background

This paper describes the development of culturally-appropriate family-based interventions to promote family health, happiness and harmony in Hong Kong. Programs were developed and implemented in the community, using a collaborative approach between a School of Public Health and several community partners. We describe the development of the interventions and their measures and the lessons learned, highlighting both the benefits and the challenges of designing locally relevant interventions in a nonwestern culture and of academic-community partnerships. We believe that information about the development of this unique project may be of interest to the international scientific community for the following reasons. First, community-based intervention studies from nonwestern cultures are relatively rare. The importance of developing interventions from "within" a culture has been recognized as important [1], but there are few examples available that delineate the associated process and challenges. Second, academic-community partnerships are increasingly relevant, particularly in cultures where community practitioners are not trained in evidence-based approaches, and community agencies offer a route for access to the population. The difficulties of disseminating evidence-based interventions have been recognized [2], and the community-based participatory approach has been lauded [3] but is still underutilized. Finally, the development of preventive interventions that can be practically applied to large samples of the population is at the interface of public health and psychology [4,5]. This partnership has promise for the future inasmuch as there has been a recent prioritization of prevention as a direction in research in mental illness [6].

There is evidence from several indicators that the traditional primacy of family in Chinese life [7,8] is under threat. Reports of domestic violence have increased threefold in the last decade [9]. Divorce has become more common, with the number rising more than eightfold in the last 25 years [10]. Cross-border marriages have increased [11] so the family may be split between Hong Kong and Mainland China. Immigration from China has increased but recent Mainland immigrants frequently have difficulty integrating into Hong Kong society. The number of families living in three-generation households has fallen 27% in 10 years, from 11.1% to 8.1% [12]. Suicide attempts per 100,000 increased from 29.5 in 1997 to 41.6 in 2003, and completed suicide rates have risen from 7.8 per 100,000 in 1982 to 13.8 in 2009 [13].

In November 2007, a Hong Kong-based charitable foundation funded the University of Hong Kong's School of Public Health to develop and implement a program entitled: "FAMILY: a Jockey Club Initiative for a Harmonious Society" (referred to in the remainder of the paper as "The Family Project"). The Family Project's goal was to enhance three key outcomes salient to Hong Kong families, health, happiness, and harmony (3Hs), via three platforms: a cohort study to determine risk factors and causes of impairment in family function; the deployment of social marketing strategies to help enhance family relationships; and the development and testing of preventive interventions to promote family relationships. Funding was allocated to the School of Public Health for six years. This paper focuses only on the intervention component's development, and reports on the first phase that lasted approximately two years following initiation.

Guidelines provided by the granting agency for the interventions to be developed were that they should: a) focus on "wellness" and not on dysfunction, i.e., the interventions be at the primary rather than secondary or tertiary prevention levels; b) target inter-family relationships; c) be developed and grounded in local priorities; d) be conducted in partnership with community agencies with emphasis on capacity building; e) be efficient and sustainable for potential scale-up across the territory; and f) be evidence-based and/or evidence-generating using the best possible evaluation design to assess the impact of the interventions to be developed.

## Methods

### Models guiding intervention development

**Figure 1:**
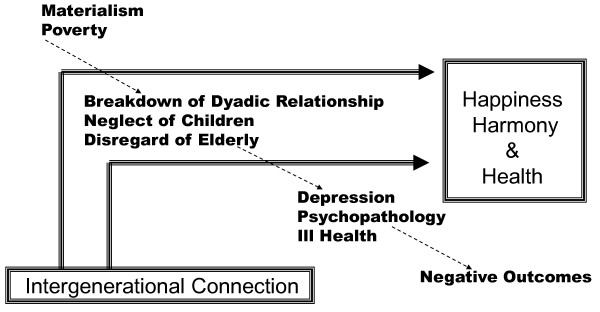
**Intergenerational connection and promotion of health, happiness, and harmony**.

**Figure 2:**
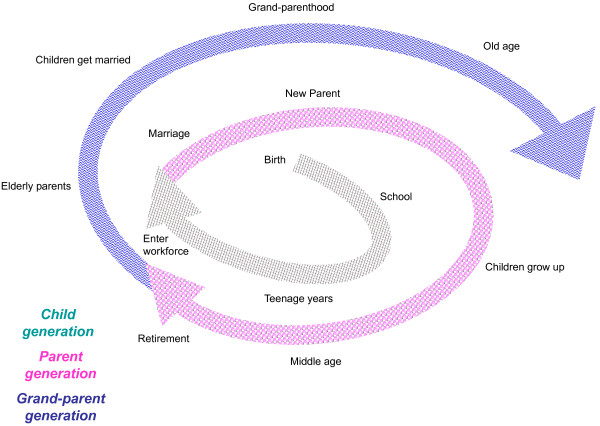
**Roles and transitions in the family life cycle**.

Over a series of meetings, the academic team developed the framework to guide intervention development. In order to develop culturally appropriate interventions, we approached the planning processes with a few guiding principles. First, that the traditional Chinese values of cherishing family relationships can be adapted to life today, and that strong family relationships will promote 3Hs for each generation of the family (Figure 1). The life cycle (Figure 2) gave a heuristic to consider changes that can affect intergenerational relationships. Transitions over the cycle usually involve significant reorganization of each family member's roles and responsibilities that often include some degree of relationship stress and emotional turmoil [14]. We saw transition points as times of vulnerability but also of opportunity to strengthen intergenerational bonds. By minimizing negative interactions and enhancing positive relationships at these times, we hypothesized that we would promote the distal outcomes of health, happiness and harmony, project-wide.

Input was solicited from overseas experts on brief, preventive interventions suitable for broad implementation. Consultant reports included recommendations for: a) clear specification of limited targets; b) clear intervention theory; c) motivational components to engage participants and enhance attitudinal change; and d) scripted manuals to allow delivery by paraprofessionals.

### The academic/community collaborative team

Interventions were developed in collaboration between the academic sector and four community non-governmental organizations (NGOs) in the social service sector, selected because they had multiple community centers in low income areas (enhancing the likelihood that we could recruit adequate numbers for our trials) and provided a broad range of services not restricted to particular demographics. Each NGO was the site for one of the four programs. A senior social worker from each NGO paired with an academic investigator to provide the leadership on the sub-team which worked on that NGO's projects from start to finish. Cross-team communication was frequent, with quarterly progress reports from all sub-teams shared among all project staff.

The academic members were primary contributors to the theoretical aspects and to the science of the project (the RCT methodology, development of assessment and quality control materials, data management and analysis), while the NGO staff brought their considerable clinical experience with the needs of the local population and their capacity for recruitment.

### Selection of targeted participants

We selected parent-child relationships as a key outcome and parents of school-age children as the target for our first interventions. This decision was made partly for practical reasons as early input from our community partners emphasized the difficulties of engaging family members other than the mother. More importantly, this focus is in line with public health priorities. The parent-child relationship is a key risk factor for problems with physical and mental health in adults [15]. Low rates of supportive parenting behaviors and high rates of harsh and negative parenting have been identified as important risk factors for mental illness [16] and poor health in adulthood [17]. We proposed that parenting behaviors that promote connection could be identified by examining the common sources of stress in the parent-child relationship.

### Strategies used to identify sources of parent-child stress

We utilized several methodologies to investigate sources of parent-child stress in Chinese culture: review of the literature; three small and informal discussion groups of convenience samples of young adult university students and research staff; and 23 groups of five to eight community parents (n = 144) organized by approximate age of their child, selected by the community agencies from their pool of community center attendees to be representative in age, education, and social status of the parents we expected to recruit into our intervention groups.

All groups were led by research staff, and participants were asked to share their view of the most significant stressors for family relationships in Hong Kong, using a series of open-ended questions. Sessions with the community groups were recorded, transcribed, and then reviewed by the research team. Themes were extracted and organized into the format that follows in the results. Data were integrated from the two discussion groups as well as the surveys, with the goal of coming to a consensus about the behaviors that were to be targeted when developing the interventions. All data collection from participants was approved by the Institutional Review Board, the research ethics committee of the University of Hong Kong.

## Results

### Themes extracted relevant to the intervention targets

The following themes emerged from the discussion groups and surveys with the Hong Kong population and guided the selection of intervention targets.

(i) Children's academic performance was an essential goal for parents, but also an important reality for access to upward mobility. Results of examinations administered from an early age determine the course of career opportunities. As a result, parent-child interaction was frequently focused on school application and preparation. Parents considered academic achievement to be an essential attribute of the "ideal child" [18].

(ii) Recent large scale surveys of the Hong Kong population elucidated stark contrasts between traditional beliefs and more individualistic goals espoused by adolescents in modern day Hong Kong [19]. Parents emphasized economic and material needs more than their children, who emphasized love, concern, support, understanding, acceptance, and togetherness more than parents [20].

(iii) The young adults in our informal groups reported high, almost unachievable parental standards for school performance, with motivation provided not with encouragement but with criticism. These young people yearned for "understanding" from their parents, but did not expect that this wish would be acceptable to their parents. Parents reported wanting to be more supportive to their children, but found that their interactions were directed largely by their children's academic needs. They found that their efforts to push their children to excel overwhelmed their efforts to be supportive.

(iv) Conflicts emerged in the parent-child relationship as the parent attempted to increase adherence to academic demands and children resisted. Parents desired help with how to motivate their child to succeed in school without scolding or battling.

(v) Parents struggled with their own emotional management. They knew that anger could interfere with their relationship with their children and they were looking for strategies to help maintain control over anger expression.

(vi) Many parents were seeking ways to manage their children's behavior in a positive way. Many had read books on parenting and even attended talks but found it difficult to apply these ideas in their own lives.

This combination of information-gathering strategies helped identify the objectives of non-punitive behavior management techniques, strategies to enhance positive parent-child interaction, and parental emotional management. The targeted changes aimed to increase "warm" parenting, and decrease "harsh" parenting, which have been linked to positive child outcomes in many cultures, including Hong Kong [e.g., [21,22]]. We hypothesized that change in these behaviors would enhance relationship satisfaction, and over time, increase family harmony, happiness and health.

### Intervention design

#### Cultural acceptability concerns

Behaviors which are interpreted as warm and harsh, and acceptable alternatives to harsh parenting can vary in different groups. Two strategies were used to ensure that the behaviors we targeted would be relevant and acceptable to the diverse samples to which we expected to offer the program. At the time of the discussion groups, we asked for information about harsh and warm parenting behaviors that are common for Hong Kong parents. Some of these behaviors became our primary outcomes. Second, the interventions adopted a large group problem-solving approach [23], described further in the sections below. This strategy ensured that specific alternatives to harsh behaviors would be generated by the parents themselves, and therefore would be more likely to be respectful of the cultural context than solutions provided by an expert with a theoretical knowledge base but limited experience with the cultural context.

#### Theoretical model

Several of the interventions built upon the Health Action Process Approach model (HAPA) [24]. This behavior change model emphasizes the distinction between the pre-intentional motivation process that drives a person's behavioral intention and a post-intentional volition process that facilitates their adoption and longer-term maintenance of the specific behavior change [24]. In both processes, *self-efficacy*, or the belief that one is capable of achieving a desired goal, is a key to initiating and maintaining behavior change. This model guided our focus on the two essential components of change: *intention *to make the desired change, and *planning*, or preparing in detail for the behavior change to move the participant from intention to action.

The model is supported by evidence from naturalistic studies from communal cultures [25-30], and from a brief intervention based on HAPA in Chinese groups [31]. In addition, this model has a number of qualities that make it particularly promising for large scale public health interventions developed in academic-community partnerships. It leads directly to brief interventions that can be easily scripted and do not require extensive training or supervision. Furthermore, it is parsimonious and highly acceptable to community partners who found it easy to understand and intuitive to implement. It has never been used to design parenting interventions to our knowledge, and as such represents an innovative application of the model.

#### Structure of the intervention

The group problem-solving approach was used in most of the interventions. Each session was focused on a single theme, e.g., "positive discipline" which we defined as non-punitive methods of managing child behavior. Videotaped scenarios were used to portray common opportunities for negative interactions between a parent and a child (e.g. a parent wants the child to turn off a video game and begin homework), where the parent's behavior had some "errors" previously identified as common by parents in discussion groups (e.g. nagging or yelling). Parents were asked to identify the erroneous parental strategies and to describe the long-term impact of these strategies on the parent-child relationship, and generate alternative strategies that might work better. Then the interventionist queried the participants on the advantages of the alternative strategies. Participants were encouraged to contribute answers to each other's concerns, with the interventionist retaining the responsibility of highlighting the key components of solutions.

The challenge of engaging and retaining participants in prevention programs has been noted by others [e.g., [32,33]]. Guided by self-presentation theory [34], we asked each parent to describe why they wanted to attend the program and make a public commitment to complete the program. Such engagement strategies maximized cognitive dissonance dynamics to enhance initial motivation. Heeding that psychoeducation works poorly in preventive programs [35,36] and didactics are less effective than presentations that allow for systematic processing of the information [37] through interaction and cognitive manipulation of the content, and that parents had told us that such programs had not been useful, we internally adopted the theme "teaching without teaching," challenging ourselves in manual development to elicit rather than provide specific content. Finally, the interventions included an important positive affective component. Observers and the interventionists noted an immediate lifting effect on the atmosphere upon the introduction of the videos that demonstrated errors in parenting. These videos were the source of considerable amusement as parents recognized the parallel to themselves and their children.

We drew on the HAPA model [24] to promote the likelihood of change by enhancing both intention and planning. We enhanced intention by encouraging attributional discussion (e.g., what would be the effect on the child if parents consistently behave the way the person in the role play is behaving?), similar to strategies used in motivational interviewing [38]. The HAPA model also emphasizes that enhancing intention is not enough to result in behavior change. The individual has to be able to visualize themselves in the situation where the behavior is appropriate and have a potential script ready to perform it; these processes are described in the model as "planning". Participants engaged in role play employing a situation relevant to their own child, and demonstrating the preferred strategies generated in group discussion. Practice was emphasized with a part of the session used to plan "homework" by participants indicating how they would make changes in the following week. Home practice was reviewed in sessions, emphasizing successful components to enhance self-efficacy, as proposed by the HAPA model.

#### Targeted participants

The target groups for the intervention were all families in Hong Kong who met eligibility criteria for the different programs. The NGOs were based primarily in lower to middle class communities, and we anticipated that these socioeconomic groups would be more likely to participate in the programs. Our programs included diverse samples, including not only middle-class native Hong Kong-born parents, but also recent immigrants from China and parents from socio-economic disadvantaged groups.

#### Development and selection of outcome to assess intervention impact

Measurement of targeted outcomes was a significant challenge. First, there is a paucity of measures validated for individuals who are not in the mainstream of English-speaking western cultures. Although some measures of parenting have been translated into Chinese and used in survey research, many are too general and stylistic to measure change. Second, in community (as opposed to clinical and selected) samples, negative behaviors may be less frequently present and positive behaviors may be more common, leaving little room for change. Finally, we were aware that the majority of the participants were not familiar with the process of observing and reporting their own behaviors, and lengthy questionnaires would be burdensome.

We measured primary outcomes by simple single item measures for behaviors (e.g. "nagging") that were identified as common in Hong Kong parents; these behaviors were targeted in our interventions. Participants reported frequency for each item ("How often in the last two weeks did you nag your child?") on a 5-point response scale ranging from *never *to *always*. These kinds of items are not amenable to usual psychometric examination, but they are commonly used in HAPA-based behavior change programs [e.g., [39]]. In order to balance between allowing individuals to settle on behaviors suggested in the group problem-solving approach, but also accurately measuring change, we included both specific behavioral items that had been identified as common and recognized readily as problematic in the process groups, and umbrella items that allowed participants some leeway in behaviors that fell into that category. Examples of the first kind of item are "nagging" and "criticizing." Our brief videos which anchored sessions showcased these behaviors, and they were quickly identified as problematic by the participants. The second kind of item allowed numerous solutions to the same endpoint. "Staying calm" was identified as a goal in the process group, and measured in our assessment. The videos showed a parent who was unable to control her anger, quickly recognized as an "error" by the participants. Discussion took place around strategies to remain calm, but we measured only the endpoint of remaining calm. The use of these single item measures in early trial groups suggested that they were more likely to show change than were broader scales of warm or harsh parenting.

We supplemented measurement strategy for primary outcomes because of concerns that baseline and follow-up assessments may be associated with different degrees of self-observation. At baseline, before the behaviors were discussed, their salience and therefore the accuracy of reporting may have been different compared to that after the program. For this reason, we also asked the participants at follow-up to report their subjective assessment of change in these behaviors since entry into the program, to better capture the small movement that would be expected following a brief program.

Secondary outcomes were the distal endpoints of perceived health (participants were asked to rate their health on a 5-point Likert scale from *poor *to *excellent*), happiness (measured by the Subjective Happiness Scale which has been previously translated and used in Chinese samples [40,41]), and harmony (using an 8-item scale developed by our research team, with Cronbach's alpha reflecting internal consistency of .92 and two-week test-retest reliability of *r *= .83 in the development sample). This scale (included in the Additional file 1) showed some evidence of construct validity as it was significantly (*p *< .001) positively correlated with mental quality of life (*r = *.26) and negatively correlated with conflict with family members (*r *= -.31)

In addition to behavioral outcomes and those measuring health, happiness and harmony, we included program evaluation information from the participants regarding their response to the program's utility, their affective responses to the program, and whether they would recommend it to others. The importance of affective components in learning has been emphasized with regard to evaluating programs [42], and these additional measures allowed us to accommodate the discomfort on the part of community partners with research methodologies which are not sensitive to clinically observed change.

## Discussion

Our programs were designed on the basis of local priorities, and informed by the local literature and discussion groups with community members. The challenge of designing brief and relevant interventions guided the selection of HAPA as the theoretical model for change, and the group problem-solving approach to engage the participants and generate culturally appropriate strategies to enhance parent-child relationships. The programs were built in close collaboration with community agencies. This collaboration enhanced the programs' cultural relevance and feasibility as we had ready partners who understood the needs of the local population, and would later facilitate access to them. The partnership required cognitive flexibility on the part of both partners as the needs for scientific soundness and feasibility had to be balanced.

### Lessons learned

(i) Academic-community partnerships require not only early involvement of community partners in the development of the interventions [43], but also mutual education, explicit discussions about issues, and genuine respect for the complementary nature of skills and experience that both groups bring to the table. NGO personnel were key members of all project teams and at least one experienced member of the NGO was also a part of the core group that developed each intervention. Through the development phase, community partners' familiarity with the participants was invaluable, significantly enriching the quality and local relevance of the study.

(ii) Differences in point of views held by clinicians and academics reappear and need ongoing resolution. Community partners were concerned about the applicability of the scientific methods to their endeavors, particularly the artificiality of manualized treatment, and insensitivity of assessment to the processes that change. Academics did not adequately appreciate the burden on participants from lengthy questionnaires. The importance of practical solutions that reduced burden on participants, the process of developing sensitive measures through clinical input, and the validity of qualitative and consumer satisfaction measures in evaluating a program were important bridges in enhancing collaboration.

(iii) Cultural issues span not only the definition of the problem, but also the implementation of the solution. Some strategies for change are more appropriate across cultures than others, because they elicit culturally appropriate solutions. Although didactic programs are simple to translate and implement at little cost, they seem to be ineffective, and if designed by external experts, they may be culturally biased or irrelevant. Attributional questions (p. 17) and group problem-solving techniques ensured content that is more likely to be specific to the individual and the cultural context.

As a result of this development process, we were able to successfully complete four pilot randomized controlled trials with four different large community agencies to demonstrate the feasibility of our approach and interventions. That we developed and implemented these trials within a brief period of two years was an indicator of successful collaboration with our community partners and unique in this region. The results of these pilots will be reported in future publications.

In summary, we presented our experience regarding the development of brief programs designed to enhance positive relationships among parents and children in Hong Kong, and the development of measures to assess behavior change from these interventions. This work has public health significance because of the global importance of parent-child relationships as a risk-factor for many outcomes in adulthood, the need for culturally-appropriate interventions for nonwestern universal populations, and characteristics of the interventions developed that promote local dissemination, including minimal additional costs for delivery for community agencies. As a result of a successful partnership with community agencies we not only developed and completed culturally appropriate trials, our projects also built capacity by enhancing community clinicians' understanding and acceptance of optimal study design and the importance of evidence in practice. This report may be useful to others in the international scientific community who have an interest in developing culturally-appropriate primary prevention trials.

## Competing interests

The authors declare that they have no competing interests.

## Authors' contributions

SMS is the principal investigator (PI) of the Intervention arm of the Family Project and took the lead role in conceptualizing and drafting the manuscript. CSF is the coordinator of the Intervention arm, a PI for one of the projects and contributed to the conceptualization and drafting of the manuscript. MH is a PI for one of the projects and contributed to drafting the manuscript. THL is the PI of the Family Project, an advocate for RCTs and helped to conceptualize and draft the manuscript. All authors read and approved the final manuscript.

## Pre-publication history

The pre-publication history for this paper can be accessed here:

http://www.biomedcentral.com/1471-2458/12/106/prepub

## Supplementary Material

Additional file 1 **Family Harmony Scale**. Click here for file

## References

[B1] Gergen KJ Gulerce A Lock A Misra G Psychological science in cultural context Am Psychol 1996 51 496 503

[B2] Klesges L Estabrooks PA Dzewaltowski DA Bull S Glasgow RE Beginning with the application in mind: designing and planning health behavior change interventions to enhance dissemination Ann Behav Med 2005 29 66 75 10.1207/s15324796abm2902s_10 15921491

[B3] Cashman SB Adeky S Allen AJ III Corburn J Israel BA Montaño J Rafelito A Rhodes SD Swanston S Wallerstein N Eng E The power and the promise: working with communities to analyze data, interpret findings, and get to outcomes Am J Public Health 2008 98 1407 1417 10.2105/AJPH.2007.113571 18556617 PMC2446454

[B4] Spijkers W Jansen DC de Meer G Reijneveld SA Effectiveness of a parenting programme in a public health setting: a randomised controlled trial of the positive parenting programme (Triple P) level 3 versus care as usual provided by the preventive child healthcare (PCH) BMC Publ Health 2010 10 131 136 10.1186/1471-2458-10-131 PMC2848632 20230604

[B5] Spoth R Redmond C Shin C Direct and indirect latent-variable parenting outcomes of two universal family-focused preventive interventions: extending a public health-oriented research base J Consult Clin Psychol 1998 66 385 399 9583342 10.1037//0022-006x.66.2.385

[B6] Insel TR Translating scientific opportunity into public health impact: a strategic plan for research on mental illness Arch Gen Psychiatry 2009 66 128 133 10.1001/archgenpsychiatry.2008.540 19188534

[B7] Lee PW Stewart SM Chan K Georgas J, Berry JW, van de Vijver F, Kagitçibasi C, Poortinga Y Hong Kong: transitions and return to the Motherland In Family structure and function across cultures: psychological variations 2006 Cambridge: Cambridge University Press 353 361

[B8] Traylor KL Chinese Filial Piety 1988 Bloomington: Eastern Press

[B9] Hong Kong Police Force Crime statistics in detail http://www.police.gov.hk/ppp_en/09_statistics/csd.html

[B10] Census and Statistics Department Number of divorces 2010 http://www.censtatd.gov.hk/FileManager/EN/Content_1149/T02_05.xls

[B11] Census and Statistics Department Women and men in Hong Kong: key statistics 2008 http://www.censtatd.gov.hk/freedownload.jsp?file=publication/stat_report/social_data/B11303032008AN08B0100.pdf&title=Women+and+Men+in+Hong+Kong+-+Key+Statistics&issue=2008+Edition&lang=1

[B12] Census and Statistics Department Women and men in Hong Kong: key statistics 2006 http://www.statistics.gov.hk/publication/stat_report/social_data/B11303032006AN06B0200.pdf

[B13] Centre for Suicide Research and Prevention, The University of Hong Kong Statistics http://csrp.hku.hk/WEB/eng/customized.asp

[B14] Cowan P Cowan PA, Hetherington M Individual and family life transitions: a proposal for a new definition In Family Transitions 1991 Hillsdale: Lawrence Erlbaum Associates 3 30

[B15] Faculty of Public Health of the Royal Colleges of Physicians of the United Kingdom Parenting and public health http://www.fph.org.uk/uploads/bs_parenting.pdf

[B16] Dallaire DH Pineda AQ Cole DA Ciesla JA Jacquez F LaGrange B Bruce AE Relation of positive and negative parenting to children's depressive symptoms J Clin Child Adolesc Psychol 2006 35 313 322 10.1207/s15374424jccp3502_15 16597227 PMC3152307

[B17] Stewart-Brown SL Fletcher L Wadsworth ME Parent-child relationships and health problems in adulthood in three UK national birth cohort studies European J Public Health 2005 15 640 646 10.1093/eurpub/cki049 16093299

[B18] Shek DT Chan LK Hong Kong Chinese parents' perceptions of the ideal child J Psychol Interdiscipl Appl 1999 133 291 302 10.1080/00223989909599742

[B19] Shek DT Perceptions of family functioning among Chinese parents and their adolescent children Am J Fam Ther 1999 27 303 314 10.1080/019261899261871

[B20] Shek DT Chinese adolescents and their parents' views on a happy family: implications for family therapy Fam Ther 2001 28 73 103

[B21] Stewart SM Rao N Bond MH Fielding R McBride-Chang C Kennard BD Chinese dimensions of parenting: broadening western predictors and outcomes Int J Psychol 1998 33 345 358 10.1080/002075998400231

[B22] Wang Q Pomerantz EM Chen H The role of parents' control in early adolescents' psychological functioning: a longitudinal investigation in the United States and China Child Dev 2007 78 1592 1610 10.1111/j.1467-8624.2007.01085.x 17883450

[B23] Cunningham CE Davis JR Bremner R Rzasa T Dunn K Coping modeling problem-solving versus mastery modeling: effects on adherence, in-session process and skill acquisition in a residential parent training program J Consult Clin Psychol 1993 61 871 877 8245284 10.1037//0022-006x.61.5.871

[B24] Schwarzer R Modeling health behavior change: How to predict and modify the adoption and maintenance of health behaviors Appl Psychol 2008 57 1 29

[B25] Renner B Spivak Y Kwon S Schwarzer R Does age make a difference? Predicting physical activity of South Koreans Psychol Aging 2007 22 482 493 17874949 10.1037/0882-7974.22.3.482

[B26] Renner B Kwon S Yang BH Paik KC Kim SH Roh S Song J Schwarzer R Social-cognitive predictors of dietary behaviors in South Korean men and women Int J Behav Med 2008 15 4 13 10.1007/BF03003068 18444015

[B27] Gutiérrez-Doña B Lippke S Renner B Kwon S Schwarzer R How self-efficacy and planning predict dietary behaviors in Costa Rican and South Korean women: a moderated mediation analysis Appl Psychol Health Well-Being 2009 1 91 104 10.1111/j.1758-0854.2008.01001.x

[B28] Schwarzer R Richert J Kreausukon P Remme L Wiedemann AU Reuter T Translating intentions into nutrition behaviors via planning requires self-efficacy: evidence from Thailand and Germany Int J Psychol 2010 45 260 268 10.1080/00207591003674479 22044011

[B29] Teng Y Mak WW The role of planning and self-efficacy in condom use among men who have sex with men: an application of the health action process approach model Heal Psychol 2011 30 119 128 10.1037/a0022023 21299300

[B30] Payaprom Y Bennett P Alabaster E Tantipong H Using the health action process approach and implementation intention to increase flu vaccination uptake in high risk thai individuals: a controlled before-after trial Heal Psychol 2011 30 1 10 10.1037/a0023580 21534678

[B31] Schwarzer R Cao DS Lippke S Stage-matched minimal intervention to enhance physical activity in Chinese adolescents J Adolesc Heal 2010 47 533 539 10.1016/j.jadohealth.2010.03.015 21094429

[B32] Renner B Schwarzer R Suls J, Wallston K Social-cognitive factors predicting health behavior change In Social psychological foundations of health and illness 2003 Oxford: Blackwell 169 196

[B33] Slep AM Heyman RE Public health approaches to family maltreatment prevention: Resetting family psychology's sights from the home to the community J Fam Psychol 2008 22 518 528 18729666 10.1037/0893-3200.22.3.518

[B34] Bem DJ Self-perception: An alternative interpretation of cognitive dissonance phenomena Psychol Rev 1967 74 183 200 5342882 10.1037/h0024835

[B35] Hedweg-Larsen M Collins BE A social psychological perspective on the role of knowledge about AIDS in AIDS prevention Curr Directions 1997 6 23 26 10.1111/1467-8721.ep11512614

[B36] Larimer ME Cronce JM Identification, prevention, and treatment: a review of individual-focused strategies to reduce problematic alcohol consumption by college students J Stud Alcohol 2002 14 148 163 10.15288/jsas.2002.s14.148 12022721

[B37] Chaiken S Heuristic versus systematic information processing and the use of source versus message cues in persuasion J Personal Soc Psychol 1980 39 752 766

[B38] Miller WR Rollnick S Motivational interviewing: preparing people for change 2002 New York: Guildford Press

[B39] Luczynska A An implementation intentions intervention, the use of a planning strategy, and physical activity after myocardial infarction Soc Sci Med 2006 62 900 908 10.1016/j.socscimed.2005.06.043 16095786

[B40] Lyubomirsky S Lepper HS A measure of subjective happiness: preliminary reliability and construct validation Soc Indic Res 1999 46 137 155 10.1023/A:1006824100041

[B41] Yeung GT Fung HH Social support and life satisfaction among Hong Kong Chinese older adults: family first? European J Ageing 2007 4 219 227 10.1007/s10433-007-0065-1 PMC5546368 28794791

[B42] Cigularov K Chen P Thurber BW Stallones L Investigation of the effectiveness of a school-based suicide education program using three methodological approaches Psychol Serv 2008 5 262 274

[B43] Sullivan G Duan N Mukherjee S Kirchner J Perry D Henderson K The role of services researchers in facilitating intervention research Psychiatr Serv 2005 56 537 542 10.1176/appi.ps.56.5.537 15872160

