# Supplementary material for: Developing community-based preventive interventions in Hong Kong: a description of the first phase of the family project
Source: BMC Public Health. 2012 Feb 7;12:106. doi: 10.1186/1471-2458-12-106 (PMC3297497; doi:10.1186/1471-2458-12-106)
Supplement: Additional file 1 — Family Harmony Scale. [file 1471-2458-12-106-S1.DOC]

### Family Harmony Scale

(1=Strongly disagree, 2=Disagree, 3=Neutral, 4=Agree, 5=Strongly agree)

1. My family gets along well.

2. Family members are happy to live together.

3. Generally, I am content with my family.

4. Compared with other families, we are very close to each other.

5. My family's day-to-day interactions are peaceful.

6. My family is harmonious.

7. My family functions well for all members.

8. My family is a happy place to be.
